# MicroRNAs and Their Targetomes in Tumor-Immune Communication

**DOI:** 10.3390/cancers12082025

**Published:** 2020-07-24

**Authors:** Sunglim Cho, Jesse W. Tai, Li-Fan Lu

**Affiliations:** 1 Division of Biological Sciences, University of California, San Diego, La Jolla, CA 92093, USA; limniim3324@gmail.com (S.C.); j2tai@ucsd.edu (J.W.T.); 2 Moores Cancer Center, University of California, San Diego, La Jolla, CA 92093, USA; 3 Center for Microbiome Innovation, University of California, San Diego, La Jolla, CA 92093, USA

**Keywords:** miRNA, oncomiRNA, post-transcriptional regulation, immune regulation

## Abstract

The development of cancer is a complex and dynamically regulated multiple-step process that involves many changes in gene expression. Over the last decade, microRNAs (miRNAs), a class of short regulatory non-coding RNAs, have emerged as key molecular effectors and regulators of tumorigenesis. While aberrant expression of miRNAs or dysregulated miRNA-mediated gene regulation in tumor cells have been shown to be capable of directly promoting or inhibiting tumorigenesis, considering the well-reported role of the immune system in cancer, tumor-derived miRNAs could also impact tumor growth through regulating anti-tumor immune responses. Here, we discuss howmiRNAs can function as central mediators that influence the crosstalk between cancer and the immune system. Moreover, we also review the current progress in the development of novel experimental approaches for miRNA target identification that will facilitate our understanding of miRNA-mediated gene regulation in not only human malignancies, but also in other genetic disorders.

## 1. Introduction

MicroRNAs (miRNA) are small, non-coding RNA molecules (~22 nucleotides) which play crucial roles in post-transcriptional regulation of gene expression. To date, more than 2600 mature human miRNAs have been registered at miRbase (Release 22.1: Oct. 2018) [1]. These mature miRNAs, incorporated together with Argonaute protein (Ago) to form the RISC (RNA-induced silencing complex), repress the expression of their targets by either inducing mRNA degradation or translational inhibition [2]. Since the first miRNA lin-4 was discovered in *Caenorhabditis elegans* in 1993 [3], miRNAs have been shown to control diverse biological pathways such as cell development, division, proliferation and differentiation, in both physiological and pathological conditions [4]. To date, many studies have reported that miRNA expression is dynamically regulated in different tumors. While dysregulation of miRNA biogenesis and function can directly contribute to tumorigenesis and malignant progression [5,6,7], considering the pivotal function of the host immune response in shaping the tumor microenvironment, the role of miRNA-mediated communication between tumors and the immune system involving exosomal miRNA, immunometabolites, and checkpoint regulators, has also begun to be appreciated. Previously, many research efforts in the identification of individual miRNA-mRNA pairs have helped shed some light on the importance of miRNAs in cancer [8]. However, as miRNAs generally repress a set of genes that are in a shared pathway or protein complex, to ensure their impact on gene regulation and the resultant biology, it is essential to obtain a comprehensive demonstration and validation of the targetome of the desired miRNAs. To this end, moving from the complete reliance on computational prediction in the early days, in the past decade, many cutting-edge experimental techniques have been developed to afford unbiased examination of the interactions between miRNA and their target mRNAs in the selected cell types [9]. These technological advances have not only made the analysis of entire targetomes for specific miRNAs possible, but also allowed us to gain mechanistic insights into the biological impact of aberrantly expressed miRNAs on tumorigenesis.

## 2. MiRNAs as Mediators of Tumor–Immune Communication

**Figure 1:**
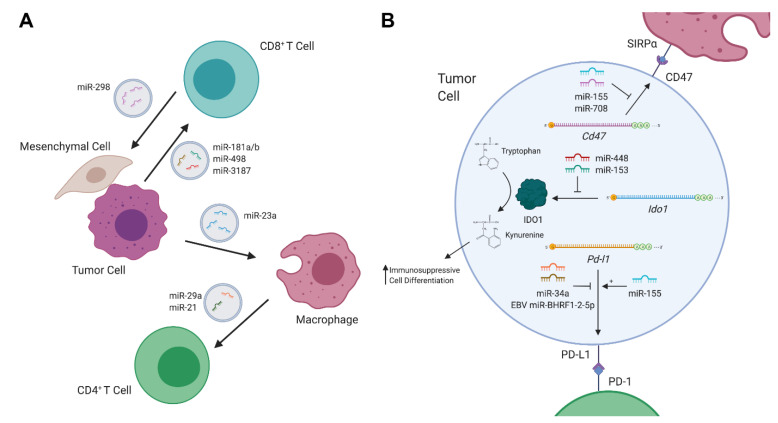
MicroRNAs (miRNAs) in tumor–immune communication. (**A**) Schematic of exosomal cross-talk between immune cells, stromal cells, and tumor cells in the tumor microenvironment (TME). (**B**) Representative diagram of miRNA-mediated control of immune-regulatory proteins in tumor cells. Created with BioRender.com.

Dysregulation of miRNAs can drive or promote carcinogenesis in a variety of fashions. In cancer, the expression of miRNAs may be dysregulated in various ways, including by mutations in the miRNA biogenesis machinery [10], changes in the epigenetic regulation of miRNA-transcribing genes [11], and altered expression of transcription factors involved in promoting or repressing miRNA expression [12,13]. Overexpression of miRNAs which target tumor-suppressive genes induce proliferative signaling, invasion and migration, resistance to apoptosis, etc. On the other hand, the loss of expression of miRNAs which target oncogenes also leads to such carcinogenic effects. It is also well known that cancer can co-opt the body’s immune system to serve its needs, whether by generating inflammation and producing genotoxic damage, or by utilizing immunosuppressive regulatory cells and molecules to evade destruction. As such, aberrantly expressed miRNAs can also be employed by tumors to communicate with, and to deactivatethe body’s defenses. Conversely, immune cells can also limit tumorigenesis through altering the expression of miRNAs in tumors (Figure 1).

### 2.1. Tumor-Derived Exosomal miRNAs

Exosomes are extracellular vesicles comprised of proteins, lipids, DNA, and RNA which are secreted by both healthy and cancerous cells. Crosstalk, mediated by exosomes between cancer cells and cells residing in the tumor microenvironment (TME), such as immune cells, fibroblasts, etc., has been implicated in driving tumorigenesis [14]. Thus, exosomes can serve as a mode of transportation by which tumor cells deliver immunosuppressive miRNAs directly to immune cell subsets (Figure 1A). To this end, a recent study found that exosomes secreted by melanoma cells altered cytokine secretion and T cell receptor signaling in CD8 T cells [15]. These melanoma-derived exosomes were enriched with miR-181a/b and miR-498, which directly bound to the 3′UTR of *TNF* and decreased TNFα secretion in CD8 T cells. Furthermore, another miRNA in melanoma-derived exosomes, miR-3187-3p, was also found to directly target *PTPRC*, a gene that encodes CD45, a key mediator of T cell receptor signaling. Such miRNAs likely serve to stymie the CD8 T cell response to melanoma and contribute to immune evasion. Notably, although the interaction was confirmed via the reporter assay, no consensus binding sequence on the *PTPRC* 3′UTR for miR-3187 was identified, highlighting the possible pitfalls of relying entirely on computational prediction.

In addition to their direct impact on T cells, tumor-derived exosomal miRNAs could also indirectly regulate T cell responses throughtargeting other immune cell subsets in the tumor microenvironment. For example, in liver cancer, miR-23a-3p released by endoplasmic reticulum-stressed hepatocellular carcinoma (HCC) cells has been reported to inhibit T cell function through targeting PTEN in macrophages from HCC tissues. Reduced PTEN expression led to elevated phosphorylated AKT, followed by increased expression of PD-L1, the ligand of PD-1 [16]. On the other hand, like tumor-derived exosomal miRNAs, miRNAs from tumor-associated macrophage (TAM)-secreted exosomes could also promote tumor growth and inhibit anti-tumor immunity. To this end, two miRNAs in TAM-derived exosomes, miR-29a-3p and miR-21-5p, have been shown to target the 3′UTR of *STAT3*, which plays a critical role in CD4 T cell differentiation into Th17 cells, thus inducing a higher regulatory T (Treg)/Th17 cell ratio [17]. It should be noted that while tumor- or TAM-derived exosomal miRNAs can suppress host anti-tumor responses, immune cells can secrete miRNA-containing exosomes to fight tumors as well. For example, Seo et al. have demonstrated that healthy, activated CD8 T cells can deplete mesenchymal stem cells (MSCs) and inhibit tumor invasion and metastasis in vivo through releasing cytotoxic extracellular vesicles [18]. The cytotoxic effect of the extracellular vesicles was attributed to miR-298-5p, a miRNA that was able to induce apoptotic depletion of MSCs, via the activation of caspase-3. This study not only highlights an anti-tumor role of immune cell derived exosomal miRNAs, but also demonstrates a novel effector mechanism by which CD8 T cells control tumor progression, independent from their canonical direct cytotoxicity against cancerous cells.

### 2.2. Immunometabolites Regulated by miRNAs

Tumors can also directly communicate with immune cells in the TME through the generation of immunomodulatory metabolites (Figure 1B). To this end, elevated expression of indoleamine 2,3-dioxygenase 1 (IDO1), an enzyme which metabolizes tryptophan into kynurenine, in the TME has been shown todrive the differentiation of several immunosuppressive cell types [19], including Treg cells [20,21,22], immunosuppressive dendritic cells [23,24] and macrophages [25,26], or to directly suppress anti-tumor immunity [27]. In colorectal cancer, overexpression of IDO1 suppressed the CD8 T cell responses, leading to enhanced tumor growth [27]. In this study, miR-448 was shown to be able to enhance the survival of CD8 T cells by directly attenuating the upregulation of IDO1 in colorectal tumor cells in response to IFNγ stimulation. Similarly, miR-153 has also been shown to target *IDO1* in colorectal cancer in response to IFNγ [27,28]. Moreover, when combining miR-153-mediated IDO1 inhibition and chimeric antigen receptor (CAR) T cell therapy, further enhanced in vitro T cell killing activities and reduced xenograft tumor growth in mice were reported [27,28].

### 2.3. Immune Checkpoint Regulators Targeted by miRNAs

Upon T cell activation, multiple co-inhibitory receptors such as CLTA4, PD-1 and LAG-3 are up-regulated. Through interacting with their corresponding ligands, these so-called immune checkpoint molecules are capable of modulating T cell responses to self proteins, as well as to tumor antigens [29]. In the past decade, great clinical success in cancer immunotherapy has been achieved by employing monoclonal antibodies targeting these immune checkpoints in patients with a variety of cancers [30,31]. In particular, therapeutic blockade of the PD-1 pathway has been considered as possibly one of the most important advances in the history of cancer treatment. Not only was PD-L1, the ligand of PD-1, found to be induced in TAM as discussed earlier, it is also highly expressed in multiple cancers [32]. PD-L1 expression can be regulated in a variety of ways, and recently the miRNA-mediated control of PD-L1 has become more apparent (Figure 1B) [33]. Indeed, disruption of the *Pdl1* 3′UTR led to elevated PD-L1 expression, implying that miRNAs play an active role in regulating PD-L1 expression [34]. To this end, recent investigation has uncovered multiple *Pdl1* mRNA-miRNA interactions in Epstein-Barr virus (EBV) associated B cell lymphomas, which are known to heavily rely on PD-L1 expression to evade immune defenses [35]. One report demonstrates that the co-localization of viral protein EBNA2, and B-cell-specific transcription factor EBF1 at the miR-34a promoter, leads to the repression of miR-34a, which targets the *PDl1* 3′UTR in Burkitt lymphoma (BL) and Diffuse Large B-Cell Lymphoma (DLBCL) cells [36]. Another group has found that a novel EBV-encoded miRNA, EBV miR-BHRF1-2-5p, could also target the 3′UTRs of *Pdl1* as well as another PD-1 ligand, *Pdl2*, in a model of EBV-positive DLBCL [37]. It should be noted that both miR-34a and EBV miR-BHRF1-2-5p coincided with LMP1 expression, which is known to drive PD-L1/2 expression. As such, the “fine-tuning” role for these counter-regulatory miRNAs may serve as attractive targets for therapeutics. On the other hand, while miR-155 expression was also higher in the serum of EBV-positive DLBCL patients [38], interestingly, miR-155-binding to the *Pdl1* 3′UTR actually served to upregulate PD-L1 expression, further demonstrating the complex nature of miRNA-mediated gene regulation [39].

In addition to the aforementioned immune checkpoint molecules that mainly act on controlling T cell responses, CD47, which is commonly expressed in blood cancers [40,41], sends a “don’t eat me” signal to macrophages, preventing them from clearing cancerous cells via phagocytosis. Like PD-L1, CD47 was also recently shown to be targeted by miRNAs (Figure 1B). Specifically, binding of miR-708 to two sites in the *CD47* 3′UTR was capable of reducing CD47 levels in T cell acute lymphoblastic leukemia [42]. Functionally, enforced miR-708 in CCRF-CEM cell lines made the cells more vulnerable to phagocytosis, an effect that was synergistically strengthened with CD47 antibodies. Moreover, CD47 was also found to be targeted by miR-155 in multiple myeloma (MM). In advanced stages of the disease, miR-155 was downregulated [43]. When miR-155 was overexpressed in drug-resistant MM cell lines, reduced amounts of CD47 accompanied with an increase in phagocytosis by macrophages were observed.

## 3. Identification of miRNA Targetome

Identifying the targets of miRNAs is essential for understanding how aberrantly expressed miRNAs in tumors or tumor-associated immune cells could impact tumorigenesis. MiRNAs of vertebrates predominantly bind to partially complementary sequences in the 3′UTR of target mRNAs. Specificity of this binding is mostly determined by 7–8 nucleotides at the 5′ end of a miRNA molecule referred to as the seed sequence. As such, many computational miRNA target prediction algorithms such as TargetScan [44], as well as PicTar [45] and miRanda [46], were initially developed by relying on the seed rule that is dictated by Watson–Crick (WC) base pairing between the 5′ end of a miRNA molecule and the conserved complementary sequences in the 3′UTR of the target mRNA. Later, it became evident that there are exceptions to the seed rule [47,48]. For example, bulges, G:U wobbles, and seedless interaction can also affect miRNA–mRNA interaction. Furthermore, strong base pairing to the 3′ end of the miRNA canalso support seed pairing and structural accessibility into target sites [49,50]. Through further integrating the various aforementioned sequence or structure criteria, the performance and the accuracy of the target prediction have improved significantly over time. Nevertheless, while these computational prediction algorithms have been widely used in miRNA research and afforded the identification of many important miRNA targets, as discussed in the previous sections, the false positive rates remain very high [51]. As such, several experimental approaches have been developed to complement existing computational target prediction methods, allowinginvestigators to gain further insights into miRNA-mediated gene regulation in cancer and other biological processes.

### 3.1. CLIP-seq

**Figure 2:**
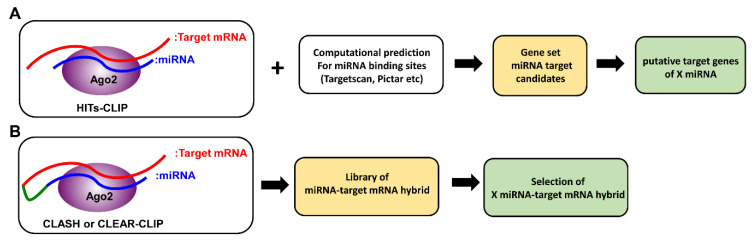
Comparison between HITS-CLIP (high-throughput sequencing of RNAs isolated by UV crosslinking immunoprecipitation) and CLASH (crosslinking, ligation and sequencing of hybrids). (**A**) In HITS-CLIP, RNA-Ago protein complexes are cross-linked with UV light and isolated by immunoprecipitation. MiRNAs and mRNAs purified from such complexes are then sequenced. MiRNA–mRNA interactions are matched computationally, and can be verified with additional assays. (**B**) In CLASH, following cross-linking, an additional ligation step generates miRNA-mRNA hybrids. Then, after isolation of RNA-Ago protein complexes by immunoprecipitation, miRNA-mRNA hybrids are sequenced to generate hybrid libraries.

CLIP-seq (cross-linking immunoprecipitation), also known as HITS-CLIP (high-throughput sequencing of RNAs isolated by UV crosslinking immunoprecipitation), is a method that was originally developed to identify functional protein–RNA interaction sites (Figure 2A) [52]. As only miRNA and mRNA incorporated into the RISC complex are pulled down for sequencing, not only does this method provide the opportunity to identify non-canonical miRNA–mRNA interactions, it has also helped exclude miRNA targets that are falsely predicted by computational means. Through taking this biochemical approach, unbiased analysis of specific miRNA–mRNA interactions has become possible [48,52]. For example, in hepatocellular carcinoma (HCC) patients, it has been previously reported that reduced expression of miR-122 in tumors correlates with metastasis and poor prognosis [53,54]. Through taking the CLIP-seq approach, many miR-122 targets, conserved in both humans and mice, and involved in thecell cycle, tight junction pathways, and cancer signaling pathways such as AMPK, PI3K/AKT, and WNT/β-catenin, were identified [53,54]. Among them, BCL9, a β-catenin co-factor which mediates transactivation of WNT target genes, was shown to be uniquely targeted by miR-122 at multiple sites. Further functional studies have established BCL9 as a conserved miR-122 target that impacts WNT-mediated progression of HCC, specifically through proliferation. Consistent with these findings, increased expression of BCL9 in tumors, along with other miR-122 targets such as STX6 and SLC52A2, are also significantly associated with poor patient survival. Similarly, like the aforementioned miR-122 in HCC, miR-203, the most highly expressed miRNA in the skin, was also found to be downregulated in the skin ofsquamous cell carcinoma (SCC) patients. Loss of miR-203 was shown to promote the initiation and development of SCC in both humans and mice [55]. Furthermore, CLIP-seq analysis revealed that miR-203 limits the proliferation of skin cells, particularly during the phase of tumor initiation, through targeting key components of the pro-proliferative Ras signaling pathway. Together, these studies demonstrate the power of the CLIP-seq approach in decoding disease-associated miRNA targeting networks and suggest that similar strategies could be applied to other tumor settings.

Building upon the basis of HITS-CLIP, another CLIP-seq method, so-called PAR-CLIP (photoactivatable ribonucleoside-enhanced crosslinking and immunoprecipitation), was established [56]. Compared to HITS-CLIP, PAR-CLIP not only offers higher crosslinking efficiency, but also allows for a more precise localization of miRNA-containing Ago binding sites. To this end, by employing PAR-CLIP analysis combined with RNA-seq in ovarian cancer cells with or without miR-450a expression, genes associated with the mitochondrial oxidative phosphorylation complex or glutamine metabolism, ACO2, TIMMDC1, ATP5B and MT-ND2, were identified as miR-450a targets [57]. Interestingly, while most of the Ago binding sites were located in the 3′UTR region, miR-450a seed complementary sequences were found exclusively in the coding sequences (CDS) of the miR-450a target genes. Nevertheless, compared to cells devoid of miR-450a, miR-450a expressing cells exhibited decreased mitochondrial membrane potential, as well as decreased glutamate production. Together, these data suggest that miR-450a regulates cellular energetic metabolism to limit tumor formation and progression.

### 3.2. CLASH (Crosslinking, Ligation and Sequencing of Hybrids)

Although CLIP-seq approaches have permitted the discoveries ofmany relevant miRNA targets in multiple studies, they havetheir own limitations [58]. Because biochemical identification of Ago binding sites came from CLIP-seq analysis of pooled mRNA after Ago immunoprecipitation, bioinformatics analysis is still required to identify the corresponding miRNAs responsible for Ago bindings. Moreover, when no obvious seed matches can be identified, there is no clear way to confirm whether the apparently seedless targeting was caused by non-canonical miRNA-target interactions or miRNA-independent mechanisms. To address this issue, building upon the original CLIP-seq approach, a new method, which is referred to as CLEAR (covalent ligation of endogenous Argonaute-bound RNAs)-CLIP [59] or CLASH (crosslinking, ligation and sequencing of hybrids) [60], was developed. In brief, miRNA–mRNA interactions are first ligated to generate miRNA–target mRNA chimeras while still bound to the Ago complex. Sequencing these miRNA–target mRNA chimeras allows for unambiguous mapping ofcanonical and non-canonical pairing (Figure 2B). Moreover, with recent advances in sequencing technology, this approach was further optimized to skip the clean-up steps that were previously required to remove unhybridized RNAs. As a result, this newly modified “quick” CLASH (qCLASH) not only is significantly faster, but can also be used to analyze patient biopsies which typically have much fewer cell numbers [61]. In this study, qCLASH was utilized to study the molecular mechanisms underlying Kaposi’s sarcoma-associated herpesvirus (KSHV)-derived miRNAs that could drive the transformation of endothelial cells. KSHV, also known as human herpesvirus 8 (HHV8), is associated with malignant tumors such as Kaposi’s sarcoma (KS), multicentriccastleman’s disease (MCD), and primary effusion lymphoma (PEL) [62,63,64]. Previously, KSHV-derived miRNAs have been shown to target tumor suppressor genes such as THBS1, TP53IPN1, and YWHAE [65,66]. By taking the qCLASH approach, 1433 gene targets were discovered and were involved in the cell cycle, glycolysis, and apoptosis pathways. Interestingly, 60% of the target sequences in KSHV-infected endothelial cells identified by the qCLASH hybrid were mapped to the CDS. In contrast, the majority of target sequences in KSHV-infected B cells are located in 3′UTR regions, indicating that miRNA–mRNA interactions can be cell type-specific. Moreover, 50% of the hybrids displayed non-seed pairing interaction, supporting the utility of generating miRNA–mRNA chimeras for the discovery of new canonical and non-canonical miRNA–mRNA interactions. Taken together, combining the qCLASH assay with pathway analysis provides an unbiased opportunity to understand the role of miRNAs in cancer biology.

## 4. Conclusions

Since the initial discovery of miRNAs back in the late 1990s, these small non-coding RNA species have been shown to be crucial for controlling almost every aspect of biological processes. In cancer, miRNAs can act both as “oncomirs” and tumor suppressors. Moreover, tumor-derived miRNAs can also impact cancer progression through directly, or indirectly, modulating immune responses, particularly in the tumor microenvironment. To this end, exosomal miRNAs released by tumor cells can directly nullify immune responses, while exosomal miRNAs released by immune cells may serve as a novel weapon in clearing cancer-associated cells, making this an exciting field for future research. On the other hand, miRNAs can also indirectly control tumor–immune communication through regulating the expression of metabolites and immune checkpoint regulators, and so this field warrants further investigation given the growing importance of such molecules in cancer immunotherapy (Table 1). Moving forward, with continued technological advances made in miRNA target identification, functional miRNA–target mRNA interactions revealed by future studies will undoubtedly provide further mechanistic insights into the development of novel anti-cancer therapeutics.

## Figures and Tables

**Table 1:** MiRNAs and their targets in tumor-immune communication.

miRNAs	Target Genes	Function	Cancer Type	Expression	Reference
miR-181a/b, miR-498	TNFα	Reduces the cytotoxicity of CD8 T cells	Melanoma	Exosomes	[15]
miR-3187-3p	PTPRC	Inhibition of TCR signaling			
miR-23a-3p	PTEN	Induces PD-L1 expression in macrophages	Hepatocellular carcinoma		[16]
miR-29a-3p, miR-21-5p	STAT3	Induces a higher Treg/Th17 cell ratio	TAM (Tumor-associated macrophages)		[17]
miR-298-5p	Unknown	Induces apoptosis of MSCs via Caspase-3	Fibrosarcoma		[18]
miR-448	IDO1	Enhance CD8 T cell survival	Colorectal cancer	Tumor cells	[27]
miR-153					[28]
miR-34a	PDL1	Blocks the PD-1 pathway	Burkitt lymphoma, DLBCL		[36]
miR-BHRF1-2-5p	PDL1, PDL2		EBV-positive DLBCL		[37]
miR-708	CD47	Promotes tumor cell elimination by phagocytosis	T-ALL		[42]
miR-155			MM		[43]
miR-122	BCL9, AMPK, PI3K/AKT, Wnt/β-catenin	Inhibits metastasis and proliferation	Hepatocellular carcinoma		[53,54]
miR-203	POLA1, HBEGF	Limits proliferation of skin cells	Squamous cell carcinoma		[55]
miR-450a	ACO2, TIMMDC1, ATP5B, MT-ND2	Limits tumor formation and progression	Ovarian cancer		[57]
